# Introduction to the Special Issue: Foods of Plant Origin

**DOI:** 10.3390/foods8110555

**Published:** 2019-11-06

**Authors:** Yasmina Sultanbawa, Michael E. Netzel

**Affiliations:** Queensland Alliance for Agriculture and Food Innovation (QAAFI), The University of Queensland, Coopers Plains, QLD 4108, Australia; y.sultanbawa@uq.edu.au

**Keywords:** plant food, composition, nutrients, vitamins, phytochemicals, fibre, processing, preservation, functional properties, health

## Abstract

Plant food is usually rich in health-promoting ingredients such as polyphenols, carotenoids, betalains, glucosinolates, vitamins, minerals and fibre. However, pre- and post-harvest treatment, processing and storage can have significant effects on the concentration and composition of these bioactive ingredients. Furthermore, the plant food matrix in fruits, vegetables, grains, legumes, nuts and seeds is very different and can affect digestibility, bioavailability, processing properties and subsequently the nutritional value of the fresh and processed food. The Special Issue ‘Foods of Plant Origin’ covers biodiscovery, functionality, the effect of different cooking/preparation methods on bioactive (plant food) ingredients, and strategies to improve the nutritional quality of plant food by adding other food components using novel/alternative food sources or applying non-conventional preparation techniques.

It is now well accepted that the consumption of plant-based foods is beneficial to human health. Fruits, vegetables, grains, nuts, seeds and plant derived products can be excellent sources of minerals, vitamins and fibre, and have usually a favourable ‘nutrient:energy ratio’. Furthermore, plant foods are also a rich source of phytochemicals such as polyphenols, carotenoids and betalains, with potential health benefits for humans. Many epidemiological studies have made a direct link between the consumption of plant foods and health. Human intervention studies have also shown that higher intake/consumption of plant foods can reduce the incidence of metabolic syndrome and other chronic diseases, especially in at risk populations like obese people. In addition to its health benefits, plant foods are also used as functional ingredients in food applications such as antioxidants, antimicrobials, natural colorants and improving sensory and textural properties. Thirteen quality papers, one review and twelve research papers are published in this special edition.

Nur Atirah A Aziz and Abbe Maleyki Mhd Jalil [1] reviewed the nutritional value and potential health benefits of indigenous Durian (*Durio zibethinus* Murr.), an energy-dense seasonal tropical fruit grown in Southeast Asia.

Akter et al. [2] studied the antimicrobial activity of *Terminalia Ferdinandiana* (Kakadu plum), a native Australian fruit rich in antioxidants. The presented results clearly demonstrated a strong antimicrobial activity of *Terminalia ferdinandiana* fruit and leaf extracts, and potential applications as natural antimicrobials in food preservation.

Thirty five tropical fruits and vegetables were screened for folate by stable isotope dilution assay (SIDA) and liquid chromatography mass spectrometry (LC-MS/MS) by Striegel and colleagues [3]. The total folate content varied from 7.82 µg/100 g (horned melon) to 271 µg/100 g fresh weight (yellow passion fruit). This study showed that some of the investigated tropical fruits and vegetables have the potential to improve the dietary supply of folate, which is regarded as a critical vitamin.

Phan and colleagues examined the nutritional characteristics and antimicrobial activity of Australian grown feijoa (*Acca sellowiana*) [4] and garlic (*Allium Sativum* L.) [5]. Feijoa fruit could be identified as a valuable dietary source of vitamin C, flavonoids and fibre. Furthermore, the feijoa-peel extracts showed strong antimicrobial activity against a wide range of food-spoilage microorganisms and may have the potential to be used as a natural food preservative. The distribution of bioactive compounds within garlic (clove vs. skin) was determined in the second paper of Phan et al. [5], to obtain a better understanding of the potential biological functionality of the different garlic tissues. Overall, the Australian grown garlic cultivars were rich in bioactive compounds and exhibited a strong antioxidant and antimicrobial activity. Industrial applications as a condiment and/or natural food preservative should be explored further.

The effect of traditional blanching methods on colour, phenolic metabolites and glucosinolates in Chinese cabbage (*Brassica rapa* L. subsp. *chinensis*) was investigated by Managa et al. [6], whereas Baenas and colleagues [7] studied the influence of common domestic cooking methods on the degradation of glucosinolates and isothiocyanates in novel Cruciferous foods. Both papers demonstrate that different cooking methods or practices can have a significant impact on the health-promoting compounds in these foods, and subsequently affect their nutritional quality.

Strategies to improve the nutritional quality of plant foods by incorporating other food components or using novel/alternative food sources were explored in four other papers [8,9,10,11]. Ballaster-Sanchez et al. [8] developed healthy and nutritious bakery products by the incorporation of quinoa. Tumuhimbise and colleagues [11] could improve the nutritional, functional, physico-chemical and sensory properties of orange-fleshed sweet potato flour, whereas Nochera and Ragone [10] developed a nutritious and gluten-free breadfruit flour pasta product. The retention of pro-vitamin A in different food products from new biofortified cassava varieties was the focus of the study conducted by Eyinla and colleagues [9].

The effect of non-conventional/innovative drying methods (microwave vacuum drying, instant controlled pressure drop-drying and conductive hydro-drying) on in vitro starch digestibility in three different cooked potato genotypes was assessed by Larder et al. [12]. The impact of emitting diode (LED) treatments on the functional quality of three types of fresh-cut sweet peppers (yellow, red and green) was investigated by Maroga and colleagues [13]. The authors could demonstrate that red LED (yellow and green sweet peppers) and blue LED (red sweet pepper) lights maintained phenolic compounds, important functional ingredients in sweet peppers, by increasing phenylalanine ammonia lyase activity.

We hope that this Special Issue will further promote the interest in plant food and its crucial role in a diverse, sustainable and healthy diet.
